# The Moderating Effects of Social Media Activities on the Relationship Between Effort-Reward Imbalance and Health and Wellbeing: A Case Study of the Oil and Gas Industry in Malaysia

**DOI:** 10.3389/fpubh.2022.805733

**Published:** 2022-03-18

**Authors:** Noreen Kanwal, Ahmad Shahrul Nizam Isha

**Affiliations:** Department of Management and Humanities, University of Technology Petronas, Tronoh, Malaysia

**Keywords:** effort-reward imbalance, health and wellbeing, social media activities, stress, office employees

## Abstract

**Background:**

Social media activities affect every aspect of human life, be it personal, social or professional. Previous studies have confirmed the role of social media in affecting health in terms of releasing stress and providing social support. Increased occupational health disorders and increased time spent on social media activities motivate us to investigate this phenomenon in the context of occupational health. Therefore, the objective of this study is to measure the effects of social media activities related to personal and social life as well as work-life on health and wellbeing of office employees, on their job efforts and job rewards, and in moderating the effect of effort-reward imbalance on health and wellbeing.

**Methods:**

Initially, to develop constructs related to social media activities, web-based structured interviews were conducted with five office employees working in the oil and gas industry for the last 5 years. Then, using an online questionnaire survey, data was collected from 424 office employees working in the oil and gas industry in Malaysia. Using statistical software for social science (SPSS) and Smart PLS, exploratory factor analysis and confirmatory factor analysis were conducted to identify reliability and validity (discriminant validity, convergent validity and composite validity) of the constructs. Following this, path analysis was conducted and the moderating effects were identified.

**Results:**

Social media activities related to work-life decrease health and wellbeing by 11% and weaken the negative effect of effort-reward-imbalance on health and wellbeing by 17.6% at a 1% level of significance. The results of social media activities related to personal and social life strengthen the negative effect of effort-reward imbalance on health and wellbeing by 12% and negatively affects health and wellbeing and job rewards by 13, 55%, respectively. The direct effect of effort-reward imbalance and job efforts is significantly negative on health and wellbeing by 59 and 10%, respectively.

**Conclusion:**

It is concluded that social media activities of the office employees significantly moderate the effect of effort-reward imbalance on health and wellbeing and intervene in job rewards in the organizations. Hence, the effect of social media activities reduces the health and wellbeing of office employees.

## Introduction

A healthy workforce is critical for organizational productivity and sustainability. Psychosocial hazards are the biggest concern for the health and wellbeing of employees worldwide. According to an Alternative International Assignments (AIA) survey (1), more than 50% of Malaysian employees suffer from work-related stress. Among them, 7% experience moderate to high levels of anxiety, and most of them are aged 18 to 40 years. Referring to the effort-reward imbalance (ERI) model proposed by (2), workers employ great effort but receive little reward and this is stressful for them; this imbalance leads to strain and long-term health concerns, such as hypertension (3, 4).

According to Rugulies, Aust (5), the effort-reward imbalance is the leading cause of mental health disorders. Several studies have identified the effects of effort-reward imbalance on health outcomes, such as sleep disturbance and fatigue (6, 7), suicidal ideation (8, 9), depressive mode and work-life balance (10), and diabetes and obesity (11). Similarly, Juvani et al. (12) found that among work stressors, effort-reward imbalance and injustice increase the risk of disability, while injustice itself leads to effort-reward imbalance. Several investigations have been conducted on the impact of effort-reward imbalance on health and wellbeing (i.e., diabetes, depression, suicidal ideation, hair cortisol, psychological health, and physiological health) (13, 14), but the factors causing effort-reward imbalance have been scarcely investigated. Among them, Heckenberg et al. (15) investigated employee traits, and found that employees with the mindfulness trait are less prone to stress; while Porru et al. (16) argued that employees with over-commitment tend to get more stressed. Therefore, to reduce occupational stress, employees use different tactics, such as smoking (17), alcohol use (18), and medical leave (19, 20).

Of late, social media is being used to release stress (21–23). However, the effect of social media on coping with stress has been investigated in the setting of students and patients only (21–23). None of the studies has identified the role of social media in the context of occupational stress (effort-reward imbalance). According to global social media statistics, about 4.48 billion people are using social media actively in 2021; whereas Malaysian statistics of social media users report 86% of the population is using social media. Social media, in terms of health improvement, is being studied; it includes coping with stress (24, 25), social support (26), stress identifiers (26), and stress releasers (22). Thus, social media is a broad platform, and its effects on occupational stress, such as that caused by effort-reward imbalance, cannot be ignored.

To identify the role of social media activities by employees, the first objective of this study is to measure the effect of stress related to effort-reward imbalance on the health and wellbeing of office employees. We investigated this aspect in the context of office employees because of their online work. They spend most of their time online using laptops and androids/tablets. The second objective of the study is to identify the effect of social media activities on the job reward of office employees.

This study has two major managerial implications. By identifying the negative effect of social media on job reward, the study evinces employee's social life interference in job reward determination. Hence, job reward that is less than the job effort generate stress within employees. Consequently, adverse employee health lowers their productivity, specifically, and the organization, as a whole. However, this managerial problem can be mitigated in two ways: first, organizations can investigate their managerial decisions, which are influenced by the social media activities of employees; and second, managers can ensure necessary measures are taken to separate employee's professional life from their personal and social life. In addition, employees should be briefed about social media usage in safety training, so that their job reward is not influenced by their social media activities.

This study adds to the body of knowledge on social media activities, which can be used by other studies to measure usage of social media and to identify its impact on other relevant constructs, such as organizational performance and organizational goodwill, among others. This study identifies two new relationships: one is between social media activities and job reward, and the other is how social media activities moderate the relationship between effort-reward imbalance and the health and wellbeing of office employees. The underpinning theory in this study is the conservation of resources (COR) theory.

The remainder of the study is organized as follows: in the next section, the background of the study and research hypotheses are given. This is followed by research methodology and data analysis, with the results presented after that. Findings, discussion and conclusion are given in the final section.

## Theoretical Background and Hypotheses

**Figure 1:**
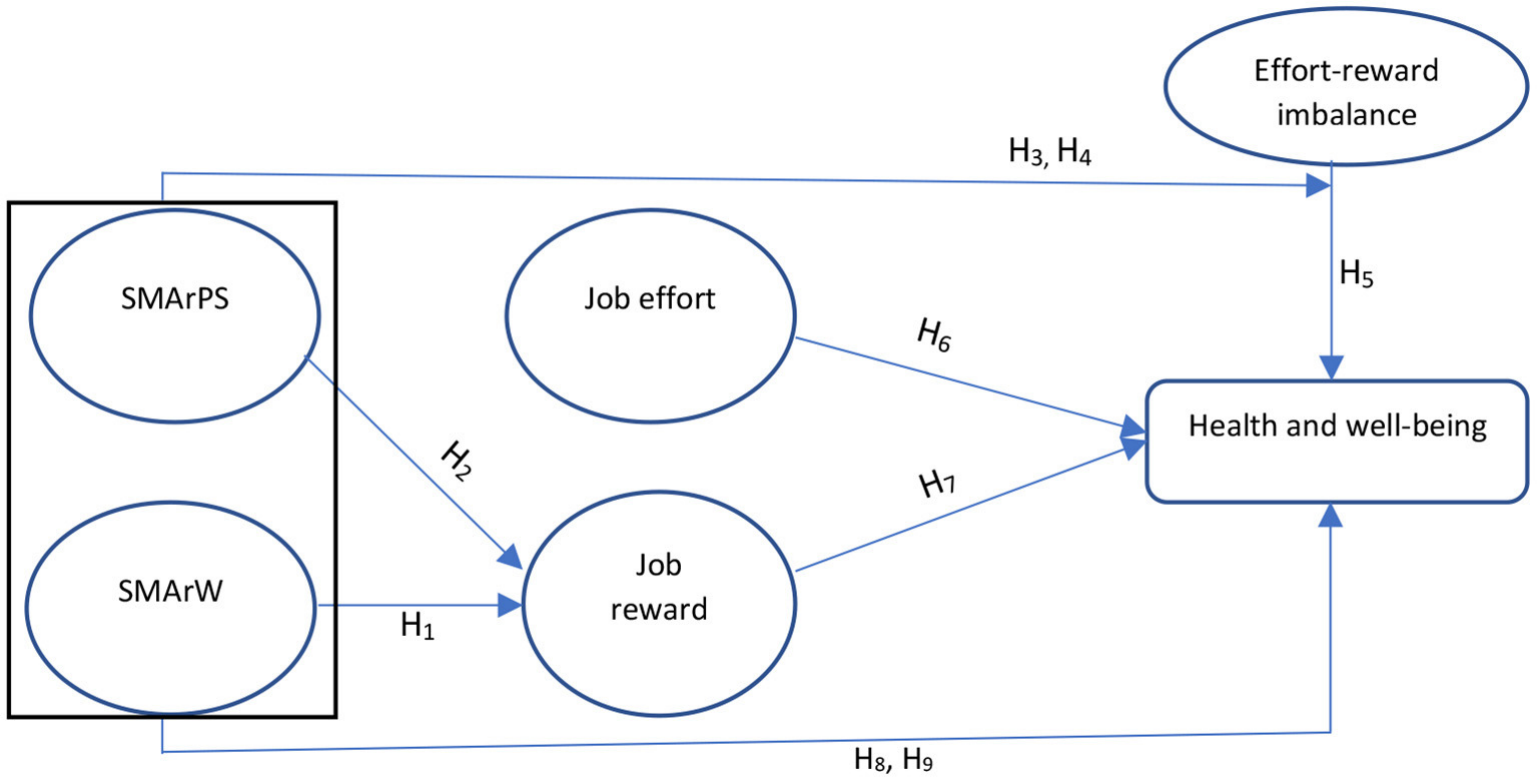
The conceptual framework: Social media activities moderating the association between effort-reward imbalance and health & well-being.

This study is based on the COR model (27, 28) that has been frequently used to explain the phenomena of stress in a range of settings (29–32). This is a resource-based model and proposes that people are motivated to acquire new resources (acquisition) in addition to retain, accumulate and preserve their existing resources (conservation). Resources are those things which people value, such as status, energy, condition and objects (33). Resources are divided into four categories (28): energy resources, such as knowledge, money and time; work resources, such as job status and reputation; material resources, such as financial and material stability; and personal resources, such as optimism as well as interpersonal resources, such as friendship and feeling valuable to others (34). This theory supposes that individuals select resources appropriately in order to maximize existing resources and to avoid future loss (27, 28, 35). Several studies have successfully applied the COR theory (36–38). For example, Halbesleben and Bowler (39) demonstrated that a higher stressor generates higher value to obtain resources in which social support is obtained through social exchange. Similarly, individuals acquire social support through social media forums, specifically in response to stress (40). According to the COR theory (41), individuals are goal-oriented and have the motivation to acquire resources, likewise, employees are motivated in the organizations to please their superiors / supervisors to gain job rewards, to get relaxation in job efforts, to initiate and maintain relationships, status building, and to release job stress (42, 43). George et al. (44) evinced that social media networking helps individuals to release stress. Specifically, they turn to social media in their down-time (stressed due to effort-reward imbalance). They disclose about themselves on social media (45, 46) for social acceptance and to get sympathy/suggestions (resources) (47). These acquired resources can generate new rewards (resources) as well as replenish expected job rewards (existing resources) (48). Therefore, COR theory supports in modeling the effect of social media activities (acquired resources) on job effort and rewards imbalance (ERI), and in moderating the relationship between ERI and health and wellbeing. Further, it is also concluded that individuals conserve and acquire resources through social media activities and these resources affect their job rewards. The proposed model of this study is as in Figure 1.

### Research Hypotheses

Evidence suggests that social networking sites (SNS) is altering social dynamics at both the micro- and macro-levels, with both online and offline consequences (49, 50). Because online social networking has become so prevalent, SNS users report a higher level of emotional support and camaraderie than average Internet users. Social media activities include self-disclosure; expression of feelings, emotions, opinions, anger and happiness; reacting to other social media posts; and reacting to other comments via likes, dislikes and complements (51–55). Social media activities are part of one's private life and individuals use these activities to gain social support and conserve resources (i.e., social capital, social status, and image building through self-disclosure, to gain information etc.). Users engage in social media activities related to personal, social and work life, according to the COR theory (29, 32, 56). In previous studies, it has been found that individuals use social media disclosure to gain social rewards in the form of gratifications and satisfaction (57, 58); and extrinsic and intrinsic rewards (49, 59). Furthermore, social media networking and activities affect peers / colleagues' behavior (60), which consequently affect superior's decisions related to job reward (61). Therefore, it cannot be concluded that user's intention to increase job rewards will actually increase rewards. This depends on user's COR since the theory proposes that individuals lacking in resources are vulnerable to experiencing loss spirals, whereas those with a lot of resources gain even more resources (48). Loss spirals as explained Sayre et al. (62) and Hobfoll (40), occurs when individuals expand resources but these resources are not available to cope with future loss threats. Therefore, in line with previous findings, in this study, we hypothesize that:

H1: Social media activities related to work life affect job reward.

H2: Social media activities related to personal and social life affect job reward.

Job reward lesser than job efforts, lead to effort-reward imbalance (63). Consequently, effort-reward imbalance generates stress (2). To release this stress, in their down-time, firstly, employees seek support from social media (58, 64–66). Through social media activities, individuals make comparisons (67). Subsequently, if individuals find themselves better than others, they feel satisfied, but if they determine their worth as lower than others, they get more depressed (68, 69). Secondly, individuals release stress by sharing their feelings of frustration and suffering on social media networks (70), and get sympathy and suggestions from social media friends (71). Accordingly, they reduce their job efforts, but in some cases, job efforts adversely affect health and wellbeing (72). This shows that social media activities moderate the effects of effort-reward imbalance on health and wellbeing. Therefore, we hypothesize that:

H3: Social media activities related to social and personal life significantly moderate the relationship between effort-reward imbalance and health and wellbeing.

H4: Social media activities related to work life significantly moderate the relationship between effort-reward imbalance and health and wellbeing.

H5: Job effort-reward imbalance has a significantly negative impact on health and wellbeing.

H6: Job effort has a significantly negative effect on health and wellbeing.

H7: Job reward has a significantly positive effect on health and wellbeing.

On the probability of getting benefits or losses from social media activities, the COR theory proposes its effect on health and wellbeing (40). Social media activities involve social comparisons (67, 73–76). The social comparison theory proposes that individuals determine their values based on how they stack up in comparison to others (76). If individuals find themselves deprived of resources, such as income and health during social comparisons, it will have an adverse effect on their health (77). Pham-Kanter (78) argued that during social comparisons, individuals find their relative position and this relative position affects health and wellbeing; it has also been found that very low position leads to cardiovascular morbidity and ulcers; while very high position decreases the probability of hypertension (78). Bao et al. (79) found negative effects of social media on wellbeing. Heidrich and Ryff (80) evinced that the effect of social comparison on health depends on upward and downward comparisons; further, they added that more frequent social comparisons worsen health. Hence, it is hypothesized that:

H8: Social media activities related to work affect health and wellbeing.

H9: Social media activities related to personal and social life influence health and wellbeing.

## Research Methodology

To achieve the objectives of the study, quantitative research method was used mainly. Therefore, the data was collected through an online survey using a questionnaire. The respondents were approached by online mode through LinkedIn application and emails. But beforehand, in-depth interviews were conducted in order to develop new measurement items and constructs used in this study. Measurement items were developed and the questionnaire was constructed following the guidelines provided by Dillman (81) and Hinkin (82). To measure health and wellbeing, a scale developed by VanderWeele (83) was adopted and extended by adding four items: (i) If you have sleeping disorders, how would you rate it?; (ii) If you have difficulty with remembering, how would you rate it?; (iii) If you have concentration problems, how would you rate it?; and (iv) If you have experienced stomach disorder, how would you rate it? Four items related to mental health were adopted from the questionnaire of Copenhagen psychosocial questionnaire (Version III): (i) If you experienced neck/shoulder pain, how uncomfortable is this?; (ii) If you have experienced lower back pain, how uncomfortable is this?; (iii) If you have experienced eye strain (blurred vision/headache), how uncomfortable is this?; and (iv) If you have experienced leg pain, how uncomfortable is this?.” Items related to musculoskeletal health were adopted from a questionnaire from Cornell university (84) and measured on a scale ranging from 0 to 10.

Items related to effort-reward imbalance were adopted from Siegrist, Starke (63). Four items for job effort and seven items for job reward, measured on a 5-point-likert scale, ranging from strongly disagree to strongly agree, were adopted from Siegrist et al. (63). Effort-reward imbalance was calculated according to the formula: e/(r × c), where c is the ratio of the number of items (here: 4/7) (63, 85).

For items related to social media activities, a survey instrument was prepared to measure the following two dimensions of social media activities of employees: social media activities related to personal life; and social media activities related to professional life, based on a comprehensive review of literature. Initially, in-depth interviews were conducted with five office employees from the oil and gas industry in Malaysia. The five interviewees were selected based on their 5 years' experience as office employees in the industry. They provided information based on their actual knowledge and experiences. Then, 12 items were developed based on interviews and literature review.

For content validity, the questionnaire was sent to three industry experts, two policy experts and three academic experts. Based on the expert's evaluation, item content validity (I-CVI) and scale content validity (S-CVI) were calculated and the results were favorable as each item was found to be valid by obtaining a score >0.78 (86). Content validity was 0.94 for relevancy and 0.98 for simplicity, i.e., greater than the standard criteria of 0.90 recommended by Polit and Beck (86).

Then, for content validity, the questionnaire was pilot tested by collecting data from 70 office employees selected randomly. After removing responses with missing values, 66 responses were used to test for reliability of the questionnaire through Cronbach' alpha via SPSS. Reliability of the questionnaire was confirmed through Cronbach's alpha of ≥0.75. After this, stratified random sampling technique was used to collect data from 471 office employees in small, medium and large companies, who carried out operations in exploration, production and development.

Responses with missing values and same responses (neutral for each question) were removed from the data. The remaining 424 responses were used for further data analysis. Normality of the data was tested through skewness and kurtosis. Herman's single factor test was used for common method bias (CMB). CMB basically occurs in survey research when all data (independent variables, dependent variables and mediating and moderating variables) are collected using the same method (87, 88). Data free from CMB is necessary for accuracy of the results; otherwise, it can bias the reliability and validity of the measures (89), as well as the estimates of the effects in regression (90).

Exploratory factor analysis was then used to reduce the summarized information contained in the observed variables of health and wellbeing and social media activities, and to identify theoretically meaningful constructs (91, 92).

The factor structure derived from exploratory factor analysis (EFA) was used to specify the measurement model using confirmatory factor analysis (CFA). CFA is a statistical technique which is used to verify the factor structure of a set of observed variables (93, 94). CFA was carried out using SPSS and smart PLS. According to Hubley and Zumbo (95), construct validity comprises convergent and discriminant validity. Therefore, convergent validity was assessed using average variance extracted (AVE), i.e., total of all standardized factor loadings divided by the number of items in each factor (96). Discriminant validity was assessed using the heterotrait-monotrait (HTMT) ratio as suggested by Henseler et al. (97). Finally, through sequential regression, the effect of social media activities on job reward and the effect of job reward on health and wellbeing were measured. Further, the moderating effect of social media activities on the relationship between effort-reward imbalance and health and wellbeing was tested.

## Results

### Demographic Results

The sample of this study comprises 424 office employees working in the oil and gas industry in Malaysia. Their demographic profile is presented in Table 1. Male respondents constitute 66.7% and female respondents comprise 33.3%. About 97.6% of the respondents are Malaysian nationals, and the remaining (2.4%) are from other countries. As for age group, more than 70% of the respondents are 31 to 50 years. Of this, 41.5% are from the age group of 31 to 40 years; 30.7% are in the 41 to 50 years age group; 17.5% are in the 21 to 30 years age group; and 10.4% are from the age group of 51 to 60 years. Statistics of education show that respondents with bachelor's degree constitute 68.4% and 21% have a master's level of education, while 4.5% have a PhD degree, 2.1% are diploma holders, and 4% have different levels of education, such as foundation and professional qualifications. In terms of job experience, more than 70% of the respondents have more than 8 years, and 12.5% have job experience of 2 to 4 years. Based on the designation, executives account for 41.3% of the study sample; 36.1% are middle managers; 20.8% are top managers; and non-executives comprise 1.9%. About 90.6% of the respondents are permanent employees; and 9.4% work on a contract basis.

**Table 1:** Respondent's demographic characteristics.

**Demographic**	**Frequency**	**Percentage**
**Gender**		
Male	283	66.7
Female	141	33.3
**Nationality**		
Malaysian	414	97.6
Others	10	2.4
**Age**		
21–30 years	74	17.5
31–40 years	176	41.5
41–50 years	130	30.7
51–60 years	44	10.4
**Education**		
Diploma	9	2.1
Bachelor degree	290	68.4
Master degree	89	21.0
PhD	19	4.5
Others	17	4.0
**Job experience**		
<2 years	4	0.9
<2 to 4 years	53	12.5
<4 to 6 years	35	8.3
<6 to 8 years	34	8.0
<8 years	298	70.3
**Designation**		
Top manager	88	20.8
Middle manager	153	36.1
Executive	175	41.3
Non-executive	8	1.9
**Job status**		
Permanent	384	90.6
Contract	40	9.4

### Exploratory Factor Analysis Results

EFA was used to understand the factor structure and for item reduction (98). Initially, 21 items were used for the factor structure for the health and wellbeing construct (Table 2). This resulted in six factors but three items were interdependent. Therefore, the three items related to the factor of general health were removed and EFA was conducted again using varimax rotation (98) based on eigen value of >1. Consequently, 19 items with six factors (happiness and life satisfaction, character and virtue, close social relationship, mental health, musculoskeletal health, and financial and material stability) were extracted; the factor loading for each item was >0.40 (99, 100). Kaiser-Meyer-Olkin (KMO) and Barlett's test of Sphericity value of 0.78, which is >0.5, indicates the sample is adequate to conduct EFA (101), which was conducted for the social media activities construct. For this, two factors (social media activities related to personal and social life (five items), and social media activities related to work life (five items), were extracted based on eigen value >1 (Table 3). Factor loading was > 0.40 and the sample was adequate to conduct the analysis as the value of KMO and Barlett's test was >0.50 (Table 4).

**Table 2:** Factor loadings by EFA of Health and wellbeing.

**Domain**	**Items**	**1**	**2**	**3**	**4**	**5**	**6**
Happiness and life satisfaction	Overall, how satisfied are you with life as a whole these days?	0.853					
	In general, how happy do you usually feel?	0.841					
Character and virtue	I always act to promote good in all circumstances, even in difficult and challenging situations.		0.801				
	I am always able to give up some happiness now for greater happiness later.		0.739				
Close social relationships	I am content with my friendships.			0.899			
	I am content with my relationships.			0.870			
	My relationships are as satisfying as I would want them to be.			0.858			
Mental health	If you have sleeping disorders, how frequently do you experience it?				0.851		
	If you have difficulty with remembering things, how frequently do you experience this?				0.803		
	If you have concentration problem, how frequently do you experience this?				0.694		
	If you experienced eye strain (Blurred vision/headache), how frequently you suffer?				0.668		
Musculoskeletal health	If you experienced neck pain, how would you rate it?					0.925	
	If you experienced upper back pain, how would you rate it?					0.897	
	If you experienced lower back pain, how would you rate it?					0.871	
	If you experienced legs pain, how would you rate it?					0.636	
Financial and material stability	How often do you worry about being able to meet normal monthly living expenses?						0.798
	How often do you worry about safety?						0.794
	How often do you worry about food?						0.778
	How often do you worry about housing?						0.773

**Table 3:** Factor loadings by EFA of Social media activity.

**Domain**	**Items**	**1**	**2**
Social media activities	I am an active social media user	0.547	
related to personal and	I do share about my interests on social media freely	0.806	
social life	I share my opinions about different things on social media freely	0.814	
	I do not hide my interests on social media	0.724	
	I like reactions by people on my social media posts	0.493	
Social media activities	I often share my work-related happiness on social media		0.599
related to work	I often share my work promotions and achievements on social media		0.669
	I often share work related sufferings on social media		0.847
	I share work related injustice on social media		0.856
	I keep an eye about my workmate's activities on social media		0.907

**Table 4:** KMO and Bartlett's test.

Kaiser-Meyer-Olkin measure of sampling adequacy.	0.787	
Bartlett's test of Sphericity	Approx. Chi-Square	5289.902
	df	171
	Sig.	0.000

### Confirmatory Factor Analysis Results

Factors identified through CFA were verified. For CFA, the model was fit as a reflective measurement model (Table 5). Outer loadings for all the items of >0.40, AVE of >0.50, and reliability measures of >0.70, were found as recommended by Darsono et al. (102).

**Table 5:** Results of reflective measurement model.

**Latent variables**	**Item indicators**	**Outer loadings**	**VIF**	**Cronbach's α**	**CR**	**AVE**
Social media activities related to	SMArW1	0.659	1.566	0.791	0.852	0.538
work life	SMArW2	0.724	1.808			
	SMArW3	0.862	2.438			
	SMArW4	0.61	1.487			
	SMArW5	0.786	1.465			
Social media activities related to	SMArPS1	0.872	2.978	0.93	0.947	0.78
personal and social life	SMArPS2	0.897	3.384			
	SMArPS3	0.894	3.317			
	SMArPS4	0.873	2.948			
	SMArPS5	0.881	3.112			
Job efforts	JE1	0.755	1.369	0.699	0.8	0.501
	JE2	0.676	2.091			
	JE3	0.709	2.037			
	JE4	0.688	1.077			
Job rewards	JR1	0.858	3.095	0.916	0.933	0.666
	JR2	0.75	2.014			
	JR3	0.752	1.863			
	JR4	0.819	2.291			
	JR5	0.853	3.126			
	JR6	0.845	2.93			
	JR7	0.826	2.686			
Effort-reward imbalance	ERI	1	1	1	1	1
Health and wellbeing	HW			0.905	0.90	0.509
Happiness and life satisfaction	HW1HLS1	0.931	2.41	0.867	0.937	0.882
	HW2HLS2	0.948	2.41			
Character and virtue	HW3CV1	0.891	1.635	0.768	0.896	0.811
	HW4CV2	0.910	1.635			
Close social relationships	HW5CSR1	0.869	1.781	0.797	0.908	0.831
	HW6CSR2	0.898	1.781			
	HW7CSR3	0.922	3.716			
Financial and material stability	HW8FMS1	0.848	1.99	0.863	0.907	0.708
	HW9 FMS2	0.842	2.077			
	HW10FMS3	0.880	2.344			
	HW11FMS4	0.795	1.851			
Mental health	HW12MH1	0.725	1.354	0.831	0.889	0.667
	HW13MH2	0.797	1.889			
	HW14MH3	0.885	3.184			
	HW15MH4	0.851	2.542			
Musculoskeletal health	HW16MsH1	0.898	2.396	0.812	0.889	0.73
	HW18MsH3	0.919	3			
	HW19MsH4	0.676	1.525			

#### Reflective Measurement Model

The results of the reflective measurement model presented in Table 5 exhibit the values of outer loading ranged from 0.61 to 0.92 for all the indicators of latent constructs. The outer loadings value > 0.70 indicates reliability of the items in the latent construct (103). However, the values between 0.40 and below 0.70 also considered as reliable if the deletion not leads to an increase in composite reliability and average variance extracted (103). Therefore, the items with outer loadings ranged from 0.61 to below 0.70 were not eliminated from the model because the removal was causing reduction in the overall reliability of the model.

### Reliability and Validity Results

Unidimensionality of the constructs were measured through Cronbach's alpha. As shown in Table 5, value of Cronbach's alpha is between 0.70 and 0.93. which is greater than the recommended threshold value of 0.70 (101, 104). Composite reliability measures the extent to which a set of construct item's share in measuring the construct. The threshold value for composite reliability is ≥ 0.70 (105), and the value of composite reliability in this study ranges from 0.80 to 0.94.

Discriminant validity was assessed through the HTMT ratio (97) for reflective constructs. The value of HTMT ratio should be <0.90 (106) for discriminant validity, and this study met the criteria as shown in Table 6.

**Table 6:** Hetrotrait-monotrait ratio.

	**CSR**	**CV**	**ERI**	**FMS**	**HLS**	**HW**	**JE**	**JR**	**MH**	**MSH**	**SMArPS**	SMArPS*ERI	**SMArW**	SMArW*ERI
**CSR**														
**CV**	0.614													
**ERI**	0.361	0.404												
**FMS**	0.251	0.229	0.304											
**HLS**	0.555	0.688	0.547	0.318										
**HW**	0.801	0.762	0.744	0.628	0.794									
**JE**	0.394	0.451	0.16	0.118	0.292	0.43								
**JR**	0.344	0.439	0.468	0.214	0.428	0.566	0.39							
**MH**	0.446	0.443	0.677	0.134	0.446	0.867	0.372	0.513						
**MSH**	0.159	0.103	0.545	0.091	0.193	0.638	0.137	0.279	0.578					
**SMArPS**	0.425	0.484	0.477	0.23	0.401	0.618	0.524	0.573	0.576	0.302				
SMArPS*ERI	0.12	0.038	0.069	0.058	0.109	0.135	0.102	0.122	0.083	0.116	0.237			
**SMArW**	0.313	0.239	0.052	0.125	0.165	0.27	0.505	0.141	0.16	0.081	0.358	0.164		
SMArW*ERI	0.087	0.109	0.282	0.039	0.046	0.084	0.131	0.043	0.036	0.056	0.114	0.425	0.091	

### Moderating Effect

**Figure 2:**
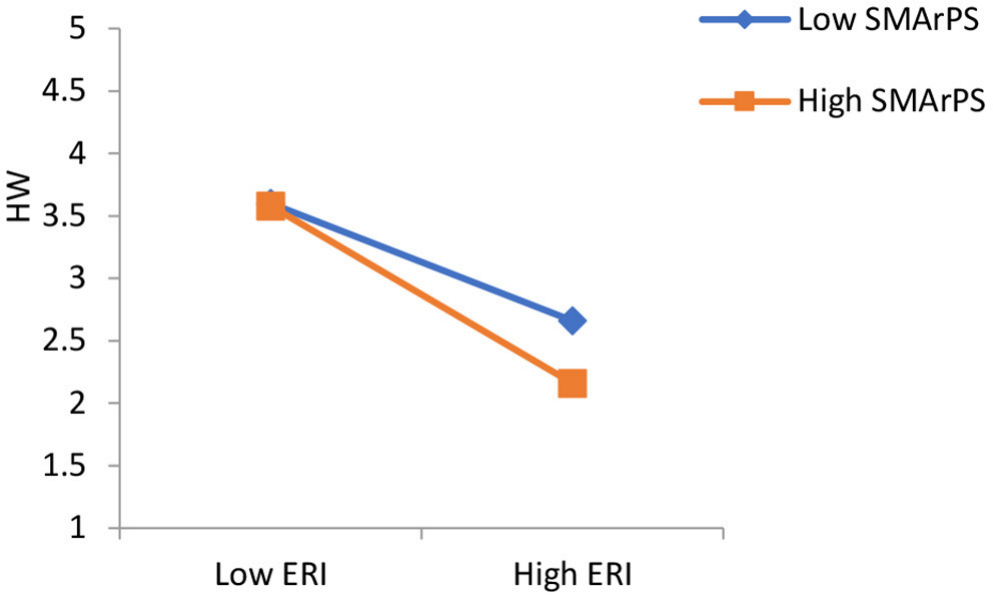
The moderating effect of SMArPS on the relationship between ERI and HW.

**Figure 3:**
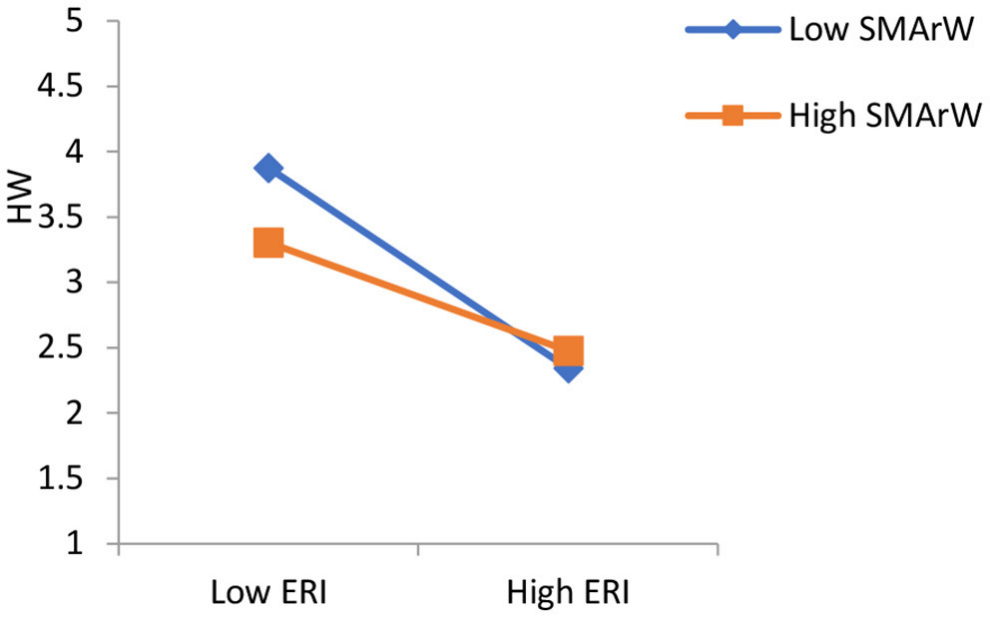
The moderating effect of SMArW on the relationship between ERI and HW.

Figure 2 shows that negative relationship between effort-reward imbalance and health and wellbeing is decreased by social media activities related to work life. Whereas, social media activities related to personal and social life strengthen the negative relationship between effort-reward imbalance and health and wellbeing (Figure 3).

### Common Method Bias

According to Podsakoff and Organ (88), CMB can occur in research if data of all the variables related to one study are collected through the same method. It can affect accuracy and robustness of the results. Therefore, to detect CMB, EFA was used, whereby all the study items were grouped into a single factor (107). It was found that one factor accounts for 14.6% of the variance, which is less than the recommended 50%. Thus, the data used for hypotheses testing in this study does not suffer from CMB.

### Path Analysis Results

The results of the structural model show (Table 7) that job reward is affected by social media activities related to work and personal and social life. Both the variables account for 28% variation in the job reward. Social media activities related to personal and social life decrease job reward by 55% at the 1% level of significance; while the effect of social media activities on work life increases job reward by 5% but insignificantly.

**Table 7:** Structural model results.

**Hypothesis**	**STD coefficient (β)**	**SE**	***R^2^***	**Results**
H_1_:SMArW → JR	0.050	0.048	0.28	Rejected
H_2_:SMArPS → JR	−0.55*	0.042		Accepted
H_3_:SMArW*ERI → HW (Moderating effect)	0.176*	0.047	0.63	Accepted
H_4_:ERI → HW	−0.59*	0.042		Accepted
H_5_:JE → HW	−0.10*	0.034		Accepted
H_6_:JR → HW	0.15*	0.039		Accepted
H_7_:SMArPS*ERI → HW (Moderating effect)	−0.12**	0.046		Accepted
H_8_: SMArPS → HW	−0.13*	0.042		Accepted
H_9_: SMArW → HW	−0.11*	0.040		Accepted

* *1% level of significant*,

** *5% level of significant*.

Job efforts significantly decrease health and wellbeing by 10%; while job reward increases health and wellbeing by 15% significantly. Effort-reward imbalance decreases health and wellbeing by 59%. Social media activities related to work increase the effect of effort-reward imbalance on health and wellbeing by 17.6% significantly; while social media activities related to personal and social life decrease the effect of effort-reward imbalance on health and wellbeing by 12% at the 5% level of significance. The R-squared value shows a 63% variation in health and wellbeing due to the independent and moderating variables.

## Discussion and Conclusion

Social media is increasingly penetrating every aspect of people's lives and affects their health and wellbeing (79), be it personal, social or professional (108). In this regard, this study evidenced intervention of employee's personal and social life into their professional lives such as effort-reward imbalance through social media activities. The study highlights the rational behavior of the employees on social media in influencing their job rewards. However, it indicates malpractices of rewarding in the organizations, which need to be addressed. Further, this study explains the behavior of the employees on social media in managing their stress related to effort-reward imbalance through online comparisons and underpinning theory of conservation of resources.

Findings from the interviews revealed social media behavior of the office employees. We find that social media has become a necessity. Employees use social media to get information (109–111) about peers, superiors, bosses and the organization. For this purpose, they follow social media timeline of their workmates and other people of interest. Related to their behavior of sharing / disclosing on social media, it is found that individuals freely share about their personal lives (happiness, sufferings and other activities) whereas they are careful in sharing about their work life. Thus, it demonstrates that individual's social media activities differ in terms of personal and work life. This is supported by the results of exploratory and confirmatory factor analysis, the tests for validating the questionnaire.

Through path analysis, we find that social media activities related to personal and social life increase the effect of stress related to job effort-reward imbalance on health and wellbeing. One of the potential reasons could be social comparisons on social media (68, 69, 112). This is because when people compare their lives with others on social media, and find themselves not better off in terms of income, reputation and enjoyment, they feel dissatisfied. Dissatisfaction increases stress and deteriorate health. On the other hand, social media activities related to personal and social life decrease job reward; this may be due to employees sharing their personal and social life and their perception of superiors on social media. This perception could be due to the personality of the employee depicted through social media, whereby the boss does not consider him or her suitable for promotion to a higher post. Another reason could be the jealousy factor raised by making his/her life's comparison with the employee's personal and social life. This affects superior's decisions in terms of obliging employees, such as granting vacation and promotion to the employee.

Social media activities related to work life reduce stress related to effort-reward imbalance on health and wellbeing. It shows that employees release stress by sharing their sufferings related to job, looking at peer's social media posts and by receiving sympathies to their own social media sharing. Therefore, through social media activities employees build personal relations to the superiors and via this unofficial forum convey their messages related to injustice in balancing job effort-rewards. Which, decreases the effects (68, 69) of effort-reward imbalance on health and wellbeing. Another reason could be the age of employees, as it has also been posited by other studies that stress among young adults is greater (113) than older adults, where more than 50% of the respondents were young adults in this study with the age group of 21 to 40 years.

Further, in this study, the results of effort-reward imbalance and job efforts affecting health and wellbeing negatively, are consistent with previous findings, such as Leineweber et al. (19), Siegrist and Li (114), Sparks et al. (115) and Haley and Miller (116). The results of the positive relationship between job reward and health and wellbeing are in line with other studies (117–120). However, in this study, we find both financial and non-financial job rewards affect health and wellbeing positively; whereas in the previous mentioned studies, only financial incentives were studied as job reward. Therefore, this study validates the findings of Giles, Becker (117); Paul-Ebhohimhen and Avenell (119); Giles, Robalino (118) and Wall, Mhurchu (120), in the context of office employees in Malaysia.

Hence, it is concluded that social media activities affect health and wellbeing of office employees as well as cause occupational stress, which arises through effort-reward imbalance; while the effect of social media activities in moderating the effect of effort-reward imbalance on health and wellbeing is different for social media activities related to work life and social media activities related to personal and social life. Further, the effect of social media activities related to personal and social life on job rewards indicates employee's private life intervenes in their professional life, which is a major risk to social freedom, and to the health and wellbeing of employees.

### Theoretical Implications

This study expands the underpinning theory of COR. Previously, it was found that social media activities, such as networking, sharing of feelings, disclosing about oneself, and relationship building, serve as an energy resource to obtain valued resources in response to stress (56, 70). However, these social media resources returned in the form of compliments negatively affect health and wellbeing of the employees. As for experiencing loss spirals (40), it occurs when resources are expanded but are not available to cope with future loss threats and can potentially lead to further loss (48). Similarly, this study identifies the increasing effects of effort-reward imbalance on health and wellbeing, moderated by social media activities related to personal and social life.

### Managerial Implications

The study findings are critical for health and wellbeing of the employees, employers and the policy makers. This study identified effort-reward imbalance which has negative effect on health and wellbeing of the office employees in oil and gas industry Malaysia. Therefore, appropriate measures should be taken to balance the job efforts and job rewards, and to improve health and wellbeing of the office employees. Moreover, management can balance the efforts and reward by reducing job efforts through improvements in job design. The negative effect of social media activities on health and wellbeing embodies problematic social media behavior of the employees, which needs to be investigated in future academic research and by the department of research and development in the organization.

Furthermore, significant moderating effect of social media activities on the relationship between effort-reward imbalance and health and wellbeing indicates intervention of employee's personal life into professional life. Such as positive effect of social media activities related work life increases job rewards. On the other hand, social media activities related to personal and social life decreases job rewards. Therefore, necessary measures should be taken by the employer to reduce the intervention of employee's personal life into work life. In this regard, firstly, the study suggests monitoring of superior's behavior in managing organizational employees at each level, especially in determining job rewards. Secondly, organizations need to ensure employee's professional life is separated from their personal and social life, so that employees can concentrate on their office work without having the tension of integrating private life into professional life. Thirdly, the subject of safe social media behavior must be included in trainings related to safety behavior in the organization, so that stress among employees generated from social media activities, for example, by social comparisons, misleading information and fake compliments, could be minimized.

The study produces the prevalent factors of psychosocial hazards in existing practices of the office employees and the management. Such as social media activities related to personal, social and professional life in effecting effort-reward imbalance and moderating its effect on health and wellbeing. Role of social media activities which is intervening personal and social lives of the employees into professional lives, can be addressed in the health and safety policies of the organizations. So, that we can remove the association of social media activities and management decisions, that is increasing the effect of psychosocial hazards (effort-reward imbalance) on health and wellbeing.

### Limitations and Future Research

This study could be enhanced in future by investigating and validating the moderating effect of social media activities related to personal, social and work life, on the relationship between effort-reward imbalance and health and wellbeing, in different cultural settings, organizations, and level of employees in the organization. This study was conducted in the context of office employees, as they have more likelihood of engaging with social media activities due to the nature of office work being online for them. Secondly, this study is cross-sectional; the results of this study can be validated in longitudinal study settings and by increasing sample size.

Further, in this study, respondents aged 21 to 40 years are vulnerable social media users. The results for older employees may be different because they are prostrate in social media networking, and this can be investigated in future for additional findings.

## Data Availability Statement

The raw data supporting the conclusions of this article will be made available by the authors, without undue reservation.

## Ethics Statement

The studies involving human participants were reviewed and approved by Ethics Committee Management and Humanities, University Technology PETRONAS, Malaysia. The patients/participants provided their written informed consent to participate in this study. Written informed consent was obtained from the individual(s) for the publication of any potentially identifiable images or data included in this article.

## Author Contributions

NK contributed in write-up, literature review, data collection, and data analysis. AI reviewed, funded, and supervised the research work. All authors contributed to the article and approved the submitted version.

## Funding

This research work is funded by YUTP research grant no. 015LC0-269.

## Conflict of Interest

The authors declare that the research was conducted in the absence of any commercial or financial relationships that could be construed as a potential conflict of interest.

## Publisher's Note

All claims expressed in this article are solely those of the authors and do not necessarily represent those of their affiliated organizations, or those of the publisher, the editors and the reviewers. Any product that may be evaluated in this article, or claim that may be made by its manufacturer, is not guaranteed or endorsed by the publisher.
